# Inflammatory Activities in Type 2 Diabetes Patients With Co-morbid Angiopathies and Exploring Beneficial Interventions: A Systematic Review

**DOI:** 10.3389/fpubh.2020.600427

**Published:** 2021-01-25

**Authors:** Martin C. Nwadiugwu

**Affiliations:** Faculty of Health and Sports, University of Stirling, Stirling, United Kingdom

**Keywords:** diabetic angiopathy, inflammation, commorbidity, intervention study, diabetes miletus

## Abstract

**Background:** Diabetes is a long-term condition that can be treated and controlled but do not yet have a cure; it could be induced by inflammation and the goal of managing it is to prevent additional co-morbidities and reduce glycemic fluctuations. There is a need to examine inflammatory activities in diabetes-related angiopathies and explore interventions that could reduce the risk for future outcome or ameliorate its effects to provide insights for improved care and management strategies.

**Method:** The study was conducted in Embase (1946–2020), Ovid Medline (1950–2020), and PubMed databases (1960–2020) using the PICO framework. Primary studies (randomized controlled trials) on type 2 diabetes mellitus and inflammatory activities in diabetes-related angiopathies were included. Terms for the review were retrieved from the Cochrane library and from PROSPERO using its MeSH thesaurus qualifiers. Nine articles out of 454 total hits met the eligibility criteria. The quality assessment for the selected study was done using the Center for Evidence-Based Medicine Critical Appraisal Sheet.

**Results:** Data analysis showed that elevated CRP, TNF-α, and IL-6 were the most commonly found inflammatory indicator in diabetes-related angiopathies, while increased IL-10 and soluble RAGE was an indicator for better outcome. Use of drugs such as salsalate, pioglitazone, simvastatin, and fenofibrate but not glimepiride or benfotiamine reported a significant decrease in inflammatory events. Regular exercise and consumption of dietary supplements such as ginger, hesperidin which have anti-inflammatory properties, and those containing prebiotic fibers (e.g., raspberries) revealed a consistent significant (*p* < 0.05) reduction in inflammatory activities.

**Conclusion:** Inflammatory activities are implicated in diabetes-related angiopathies; regular exercise, the intake of healthy dietary supplements, and medications with anti-inflammatory properties could result in improved protective risk outcome for diabetes patients by suppressing inflammatory activities and elevating anti-inflammatory events.

## Background

Diabetes is a chronic condition with rising global prevalence that is associated with multimorbidity (1). It is linked with metabolic abnormalities such as increased oxidative stress, free radicals, accumulated free fatty acids in the blood, and the onset of inflammatory changes (2). There are currently no cures for diabetes and its signs and severity differ among people of different ethnic groups (2). The economic cost of treating diabetes increases with increasing age and even more so for clinically managing the disease and its associated comorbidities (3).

The clinical treatment goal in managing people with diabetes is to prevent the onset of associated co-morbidities by relieving symptoms, and controlling hyperglycemia and hypoglycemic episodes upon diagnosis (2). According to Reutrakul and Mokhlesi (4) people living with type 2 diabetes mellitus (T2DM) constitute about 90–95% of all people living with diabetes, and they often present several associations with hypertension, cardiovascular diseases, nephropathy, retinopathy, depression, inflammatory and neurodegenerative diseases, and so on, most of which are recognized when co-morbidities becomes evident (5). About 97.5% of people with T2DM have at least one co-morbid condition while about 88.5% have two or more additional disorders (6).

Although inflammation has been associated with diabetes (2), the key question remains on finding supporting strategies that prevent worsening outcome. Inflammation is a pathological process caused by diverse cytologic and chemical reactions (7). The hallmarks of inflammation are dysfunction and injury to tissues usually manifested by loss of function, heat, pain, and swelling (7). Damage to small blood vessels (diabetic microangiopathy) and to the larger vessels (diabetic macroangiopathy) generally referred to as diabetic angiopathy is the major vascular complication of diabetes (8). Diabetic microangiopathy includes diabetic retinopathy, nephropathy, and neuropathy, whereas complications from macroangiopathy include cardiovascular events such as heart attacks, strokes, and insufficient blood flow (8). Evidence from large randomized controlled trials (RCTs) have suggested that optimal metabolic control in T2DM could delay the onset of both diabetic micro and macroangiopathy (8). Moreover, changes in metabolic status such as changes in the status of cytokines in adipose tissues could increasingly alter insulin action (9). The accrual of circulating pro-inflammatory cytokines and reactive oxygen species (ROS) in prediabetes patients have been reported to disrupt intestinal barriers and compromise tight junctions between cells allowing for deleterious compounds to be increasingly absorbed (10).

Pro-inflammatory cytokine production activates myeloperoxidase enzymes and NADPH oxidase (NOX) involved in amino acid oxidation to produce advanced glycation end-product (AGE) precursors (11). Like pro-inflammatory cytokines, AGE is a biomarker for vascular complications (12); its production within the body is accelerated by oxidative stress, dyslipidemia, and hyperglycemic conditions associated with diabetes patients (13), and is particularly elevated in people who also have impaired renal function due to decreased urinary AGE excretion (14). The ability of the receptor for advanced glycation end-products (RAGE) to interact and bind with AGEs is thought to promote atherosclerosis, endothelial dysfunction, and has an inflammatory effect in diabetes complications by inducing lipid abnormalities, oxidative stress, and macrophage uptake via upregulation of inflammatory cytokines such as IL-1, IL-6, and TNF (15–17).

The objective of the study was to systematically review literature to investigate inflammatory activities in diabetes-related angiopathies and to explore beneficial interventions, as the evidence to support strategies to successfully cope with the chronic illness of T2DM is patchy, and the possibility for worsening condition or reversal substantial (18). The overarching aim of the study was to identify

1. Inflammatory activities in T2DM-related angiopathies and
2. Beneficial interventions for reducing the risk of inflammatory activities.

The remaining sessions of this study will discuss the methodology, search strategy, and quality assessment of the systematic review. Next, inflammatory activities and T2DM-related angiopathies will be overviewed in relation with findings from the selected studies. A narrative synthesis of the effect of interventions on measures of inflammation will be included. The study will conclude by summarizing the findings and how they may be beneficial for diabetes patients with co-morbid conditions.

## Methods

### Rationale

This study reviewed only RCTs (19). Since the outcome of interest was to investigate inflammatory activities in diabetes-related angiopathies and explore beneficial interventions, articles from RCTs reporting inflammatory activities in people with diabetic angiopathies after an intervention or in comparison with an experimental group were of interest. The study was registered in PROSPERO (CRD42020204001).

### Inclusion and Exclusion Criteria

Only articles from peer-reviewed journals published in English with a focus on human subjects (18+ years) and from any country were included. The timeline of study in the selected RCTs was at least 14 days. Included studies reported the outcome of an intervention on inflammatory markers, namely, drugs, dietary supplement, aerobic exercise, as well as blood glucose levels and risk for diabetic angiopathy. The significant differences in measures of inflammation after the intervention in each RCT were reported. Animal studies were excluded. The PICO framework used to define the eligibility criteria is seen below:

P—Population: type 2 diabetes patients
I—Exposure: inflammation
C—Comparison/control: healthy individuals
O—Outcome: diabetic angiopathies

Only RCTs on type 2 diabetes reporting inflammatory activities in diabetes-related angiopathies and beneficial interventions were eligible (see Table 1). Studies involving people who do not have diabetes or other types of diabetes were excluded. The search items can be seen in Table 2.

**Table 1:** Inclusion/exclusion criteria.

	**Inclusion**	**Exclusion**	**Rationale**
Population	Type 2 diabetes patients	People who do not have diabetes or other types of diabetes will not be included	To review inflammatory activities in T2DM-related angiopathies
Exposure	Inflammation	Inflammation not linked with diabetes	To focus on inflammatory activities as well as beneficial interventions
Comparison	Healthy individuals		
Outcome	Diabetic angiopathies	Other co-morbidities that are not diabetic angiopathy	To investigate induced-risk for inflammation and strategies for improved protection

**Table 2:** Search items.

**Diabetes (type 2) (step 1)**		**Co-morbidity (step 2)**		**Exposure (step 3)**
**Terms** “Type 2 diabetes” OR “Type II diabetes” OR “Diabetes-Mellitus-Non-Insulin-Dependent” OR “diabetes type 2” OR “Adult Onset Diabetes Mellitus” OR “Slow Onset Diabetes Mellitus” OR “Adult-Onset Diabetes Mellitus” OR “Slow-Onset Diabetes Mellitus” OR “Stable Diabetes Mellitus” OR “Ketosis Resistant Diabetes Mellitus”	**AND**	**Terms** “Diabetic Microangiopathy” OR “Microangiopathies Diabetic” OR “Diabetic Microangiopathies” OR “Vascular Diseases Diabetic” OR “Angiopathies Diabetic” OR “Diabetic Vascular Complication” OR “Diabetic Vascular Disease” OR “Vascular Complications” OR “Diabetic Vascular Disease” OR “Diabetic Vascular Diseases” OR “Diabetic Vascular Complications”	**AND**	**Terms** Inflammation^*^ OR “Acute-Phase Reaction” OR “Foreign-Body Reaction” OR “Neurogenic Inflammation” OR Seroma^*^ OR “Serositis” OR Suppuration^*^ OR “Systemic Inflammatory Response Syndrome”
**Combined with OR**		**Combined with OR**		**Combined with OR**

### Search Strategy

The review was conducted in Ovid Medline, Embase, and PubMed databases. Ovid Medline is a large database of healthcare, medicine, and scientific disciplines. PubMed is a biomedical and genomic database accessed from the National Center for Biotechnology Information (NCBI) platform, whereas Embase is a widely used biomedical and pharmacological bibliographic database hosted on Elsevier. Keywords for the population, exposure, and outcome were used to search each database. Terms for the population “type 2 diabetes patients” and outcome “diabetic angiopathies” were retrieved from the Cochrane library (20), whereas terms for the exposure “inflammation” were identified using PROSPERO MeSH thesaurus qualifiers (7). The terms can be seen in the database search history (Appendix 1: PubMed Queries; Appendix 2: Ovid Medline Queries; Appendix 3: Embase Query).

**Figure 1:**
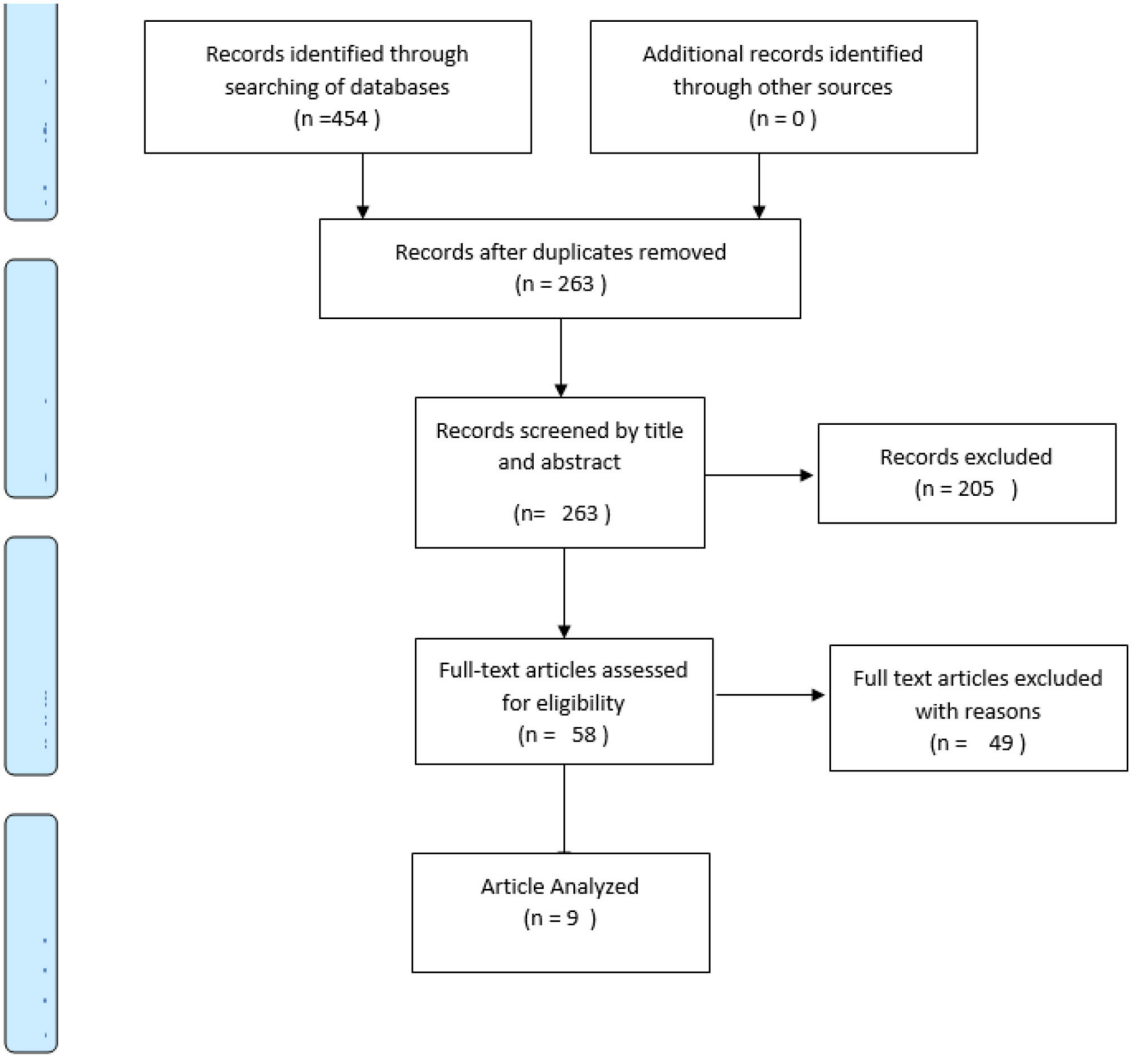
PRISMA flow diagram.

The key categories searched were diabetes (type 2), inflammation, and diabetic angiopathies. Synonyms for each key category generated were individually searched and then collectively combined with the “OR” logical operator. An intersection between the three key categories searched was done using the “AND” operator as shown in Table 2. The screening process from the three databases yielded 454 total hits and is presented in the PRISMA flow diagram in Figure 1. One hundred and ninety-one duplicates were removed after initial screening by titles and abstracts. Two-hundred and five articles were not included because they did not meet the eligibility criteria. Out of the 58 articles left, 49 were excluded for not fully meeting the selection criteria as they either only highlighted protocols or were about inflammation linked with other disorders. Nine RCTs met the inclusion criteria and were included in the narrative synthesis and quality appraisal of the findings.

### Data Extraction and Quality Assessment

Data from the nine selected studies were extracted under these headings: name of author(s), year of publication, study design, co-morbidity type, inflammatory associations, methods, and intervention as seen in Table 3. The quality assessment of the selected studies was done using the Center for Evidence-Based Medicine Critical Appraisal Sheet (21). Data about the participants, interventions used for the RCTs, objectivity of the blind study, if the groups were similar and treated equally at the start of the trial, and validity of the outcome of treatment were examined using the CEBM appraisal sheet. The validity of the outcome of treatment for each study was rated “high,” “moderate,” or “low” based on the quality (22). The criteria for rating were lack of blinding, selective outcome reporting, and incomplete accounting of outcome events based on the GRADES guidelines by Guyatt et al. (22). Studies that did not meet all three criteria were considered as “high quality,” those that met only one were considered “moderate quality” while those that met two or more were considered “low quality.” Inflammatory mechanisms from the RCTs were analyzed and used in explaining the activities of inflammation in diabetes-related angiopathies.

**Table 3:** Quality appraisal.

**References**	**Treatment**	**Participant accountability**	**Double blind**	**Treatment/outcome**	**Validity**
Alkhalaf et al. (23)	Baseline characteristics shows equal treatment	Participants were accounted for and the lack of follow-up was explained. However, they were all males in the older category which limits representation across groups	Yes	Not significant: Inflammation induced T2DM angiopathy unaffected by benfotiamine	Low quality: Incomplete accounting of outcome events and selective outcome reporting Limitation: Participants also had other medications such as angiotensin receptor blockers (ARBs), ACE inhibitors
Choi et al. (24)	Baseline characteristics show equal treatment	It was unclear why 3 participants were dropped during intervention. Only Asian women with T2DM	Not applicable	Significant: Aerobic exercise improved systemic inflammatory state in T2DM	High quality. Limitation: Comprehensive detailing and clustering of the participants based on drug types to support disclosure that other medications did not influence the findings
Homayouni et al. (25)	Baseline shows equal treatment	Participants were accounted for and the lack of follow-up was explained. Unreported or poor dietary compliance of hesperidin supplement by the participants is a limitation	Yes	Significant: Hesperidin was found to have anti-inflammatory effects in T2DM	Moderate quality: Incomplete accounting of outcome events Limitation: Unreported or poor dietary compliance of hesperidin supplement by the participants
Schell et al. (26)	Baseline shows equal treatment	Unclear why 3 participants were dropped for the crossover 4-week study. Skewed toward males (25 male participants and 5 females)	No	Significant: Daily raspberry consumption lowered IL-6 and hs-TNF-α (*p* < 0.05) Not significant: CRP levels	Moderate quality: No blinding Limitation: Short duration of study
Barzilay et al. (27)	Participants who received salsalate had a higher heart rate (74.16 10.8 vs. 71.06 9.1) and used less non-ACE/angiotensin receptor blocker hypertension medications (33.1 vs. 46.8%; *p* = 0.03) than those who received placebo	Unclear. Slight difference between participants in the intervention and control. Lack of information on number of participants during follow-up. Participants were older adults less likely to be non-whites and depend on lifestyle modification for T2DM treatment	Single masked	Mixed - Salsalate moderated hyperglycemia and serum levels of AGEs - Early AGE was reduced, late AGE was not. There was reduced HbA1c and inflammatory markers	Moderate quality: Incomplete accounting of outcome events Limitation: Change in serum-protein-bound AGE levels was not exactly known. Most participants in the salsalate-treated group also took reduced doses of antidiabetic drugs
Kadoglou et al. (28)	Baseline study did not differ	Participants were accounted for. There were more women (34) than men (26)	No	Significant: There was a reduction in hs-CRP after aerobic exercise - Anti-inflammatory mechanisms were stimulated	Moderate quality: No blinding Limitation: - The participants had no history of cardiovascular complications - The intensity of exercise for the experimental group was unclear
Krysiak et al. (29)	Baseline shows equal treatment	Participants were accounted for. There were more males (108) than females (82)	Yes	Significant: Found significant decrease (p = 0.001) in all of TNF, IL-1, IL-2, IL-6, MCP-1, and CRP inflammatory markers after a combined treatment with simvastatin and fenofibrate, but a weak correlation (r = 0.20) with single fenofibrate administration	High quality. Limitation: While there was agreement on combined simvastatin and fenofibrate administration, there were mixed results with the ACCORD trial on monotherapy
Zarezadeh et al. (30)	Baseline shows equal treatment	Participants were accounted for. Data on gender of selected participants were missing	Yes	Significant: Ginger supplementation led to a change in ADMA after intervention, but a decrease in sICAM-1 was not statistically significant	Moderate quality: Incomplete accounting of outcome events Limitation: ADMA and serum ICAM-1 differences between the study groups were not statistically significant
Mizoguchi et al. (31)	Baseline shows equal treatment	Participants were accounted for. Skewed toward males (39) than females (13)	Yes	Significant: HDL increased, while hs-CRP levels decreased after pioglitazone (p < 0.01) treatment. Glimepiride increased hs-CRP (p < 0.05)	High quality. Limitation: Small participant size, participants were on other drugs, short duration of studies but findings concurred with few similar studies

## Results

The result of the database search is presented in Table 4. From the selected studies in Table 5, the highlighted inflammatory activities were high-sensitivity C reactive protein (hs-CRP), myeloperoxidase (MPO), serum amyloid-A (sAA), tumor necrosis factor-alpha (TNF-α), interleukins (IL-1, IL-2, IL-6, IL-18, IL-10), soluble receptor for advanced glycation end-products (sRAGE), intercellular adhesion molecule-1 (ICAM-1), monocyte chemoattractant protein-1 (MPC-1), white blood cells (WBC), and total anti-oxidant capacity (TAC) (23). Hs-CRP was the most common inflammatory biomarker as it was measured by seven of the nine selected studies. This was closely followed by TNF-α (five times) and IL-6 (four times). TNF-α, IL-1β, IL-6, MCP-1, IL-2, and interferon-γ are some of the monocyte- and lymphocyte-derived pro-inflammatory cytokines (29). These cytokines including CRP are markers of chronic low-grade systemic inflammation and a prognostic indicator of vascular complications and increased cardiovascular risk (29). While IL-10 is an anti-inflammatory cytokine, IL-18 is a pro-inflammatory cytokine. The IL-18/IL-10 ratio represents the balance that foretells the incidence of coronary complications (32–34). The elevation of IL-18 promotes atherosclerosis by interferon-γ release which are balanced by IL-10 cytokines (32, 35). Thus, disequilibrium of IL-18/IL-10 ratio may promote atherosclerotic process.

**Table 4:** Database search results.

**Databases**	**Step 1 result combined with ‘OR' operator**	**Step 2 result combined with ‘OR' operator**	**Step 3 result combined with ‘OR' operator**	**Result of steps 1, 2, and 3 combined with ‘AND' operator**
Embase	123,834	9,687	1,211,662	131
Ovid Medline	130,433	12,120	538,190	104
PubMed	179,274	14,951	567,008	219
Total	433,541	36,758	2,316,860	454

**Table 5:** Data extraction.

**Author name and year**	**Study design**	**Intervention**	**Location**	**Participant**	**Duration**	**Measured inflammatory activity**	**Co-morbidity type**	**Outcome**	**Statistical significance**
Alkhalaf et al. (23)	RCTs	Benfotiamine (e) or placebo (c)	Netherlands, 1 outpatient clinic	86 males: 39 (e) 43 (c); age: 40–75 years Health status: T2DM, UAE	12 weeks	Hs-CRP, serum amyloid A (sAA), myeloperoxidase (MPO)	Diabetic nephropathy	Intervention insignificant on chronic low-grade inflammation	All *p* > 0.05
Choi et al. (24)	RCTs	Exercise (e) and control (c) groups	Korea, 1 School of Medicine	80 Females: 38 (e) 37 (c), age: 54.4 ± 6.6 years Health status: T2DM, <2 kg, sedentary lifestyle	12 weeks	sRAGE, Hs-CRP, IL-6	Vascular complications, cardiometabolic risk	Decreased hs-CRP, IL-6, waist circumference, systolic and diastolic blood pressure, HbA1c, glucose, AST, apolipoprotein B, and free fatty acid	sRAGE to Hs-CRP (*p* = 0.019; *r* = −0.27)
Homayouni et al. (25)	RCTs	Hesperidin supplement or placebo	Iran, 1 University Medical Clinic	Total: 64; Male: Female: Age: 30–65 years Health status: T2DM, BMI <30	6 weeks	TNF-α, IL-6, hs-CRP, total anti-oxidant capacity (TAC)	Vascular complications	Reduced levels of inflammatory markers	IL-6 (*p* = 0.001) and hs-CRP increased (*p* < 0.000), TAC (*p* < 0.000) decreased
Schell et al. (26)	Randomized crossover study	Dietary raspberries	USA, 1 Clinical Assessment Unit	Total: 25 Male: 5 Female: 20 Age: 54 ± 4.2 years Health status: T2DM, waist circumference (men: >102, women: >89 cm)	2 days postprandial phase 1 week apart A 1-week washout phase. 10-week “diet supplement phase” of 4 weeks with 2 weeks apart	(IL)-6 and hs-TNF-α	Vascular complications	Red raspberries significantly improved postprandial hyperglycemia and selected markers of inflammation	All *p* > 0.05
Barzilay et al. (27)	RCTs	Salsalate	USA	Total: 227 ≥75 years Male: 130 Female: 97 Health status: T2DM, HBA1c: 7–9.5	48 weeks	CRP, TNF-α, WBC types	Vascular complications	Lowered HbA1c, reduction in WBC, neutrophil, and lymphocyte counts indicated anti-inflammatory role. Changes in furosine was positive with CRP and TNF changes but not other glycation products	*p* > 0.05 for CRP and TNF for furosine changes in salsalate users
Kadoglou et al. (28)	RCTs	Aerobic training program (exercise)	Greece	Total: 60 Male: 26 Female: 34 Age: 61.64 ± 4.9 years Health status: T2DM, obese, HBA1c: 6.5–8.5, no diabetic angiopathy, postmenopausal women	6 months (four times/week, 45–60 min/session)	hs-CRP, IL-10, IL-18, TNF-α	Cardiovascular risk, without vascular complications	Exercise training improved metabolic profile and exerts anti-inflammatory effects in T2DM	Reduced CRP (*p* = 0.04) and IL-18 (*p* = 0.02). Increased IL-10 (*p* = 0.039); suppressed IL-18/IL-10 ratio (*p* = 0.014)
Krysiak et al. (29)	RCTs	Simvastatin, fenofibrate, simvastatin + fenofibrate, and placebo	Poland, 1 large Teaching Hospital	Total: 190 Age: 25–75 years Male: 108 Female: 82 Health status: untreated T2DM	180 days−90 days lifestyle modification and 90 days for the intervention	TNF, IL-1, IL-2, IL-6, MCP-1, plasma C reactive protein levels	Systemic inflammation, atherogenic dyslipidemia	Simvastatin and fenofibrate decreased monocyte release of TNF, IL-1, IL-2, IL-6, MCP-1, and lymphocyte release of IL-2, plasma C reactive protein levels. Anti-inflammatory effects of fenofibrate partly correlated with the improvement in insulin sensitivity	All significant (*p* = 0.001), but weakly correlated for fenofibrate alone on hs-CRP (*r* = 0.20 to 0.29, *p* = 0.001)
Zarezadeh et al. (30)	RCTs	Ginger powder and wheat flour	Iran, 1 large Teaching Hospital	Total: 48 Age: 30–60 years (both genders) Male: Female: Health status: T2DM b/w 1 and 10 years	10 weeks	Intercellular adhesion molecule 1 (ICAM-1)	Vascular complications, cardiovascular disease	Ginger supplementation decreased sICAM-1 and ADMA after intervention but was marginally significant	Ginger supplement decreased sICAM-1 marginally (*p* = 0.097)
Mizoguchi et al. (31)	RCTs	31 pioglitazone-treated and 21 glimepiride-treated patients. Serial ^18^F-fluorodeoxyglucose positron emission tomography (FDG-PET) imaging	Japan, 1 large Teaching Hospital	Total: 56 Age: 60–76 years Male: 39 Female: 13 Health Status: T2DM	4 months	hs-CRP	Impaired glucose tolerance, atherosclerotic plaque inflammation	High-sensitivity C reactive protein was decreased by pioglitazone, whereas it was increased by glimepiride	h-CRP levels decreased by pioglitazone (*P* < 0.01) but increased marginally by glimepiride(*p* < 0.05)

### Overview of the Findings

An overview of the findings from the nine selected studies (Table 5) shows that the interventions could be explained and grouped into three main categories: exercise, diet, and medication. Two studies (24, 28) investigated the effects of aerobic exercise on markers of inflammation (hs-CRP, sRAGE, IL-6, IL-10, IL-18) in T2DM participants with cardiovascular or cardiometabolic risk. Three studies (25, 26, 30) examined the effect of dietary supplements, while four studies (23, 27, 29, 31) investigated the use of medication as an intervention. A further overview and appraisal of each study under the appropriate category (exercise, diet, medication) is explored in the following section.

### Effect of Aerobic Exercise on Measures of Inflammation in People With T2DM

To understand inflammatory activities in T2DM, Choi et al. (24) explored the effects of exercise on inflammatory markers and soluble receptor for AGEs on 75 patients with T2DM for 12 weeks and found that, apart from the physical benefits of exercise that was observed such as a reduction in waist circumference, and loss of weight and body fat percentage leading to a reduction in risk factors for atherosclerosis and cardiometabolic diseases (e.g., decrease in blood pressure, free fatty acids, oxidative stress), there was a significant decrease in the levels of hs-CRP and an increase in soluble receptor for AGEs in the exercise group but not in the control group, which suggests that aerobic exercise improved systemic inflammatory state in T2DM. While the level of moderate intensity of the aerobic exercise may not be exactly quantified, the study found an independent association (*R*^2^ = 0.244) of age, glucose, cholesterol levels, and triglyceride with soluble receptor for AGEs (24). Although this is a weak association, it implies that aerobic exercise had a positive influence on glucose, cholesterol, and triglyceride levels. Similarly, Kadoglou et al. (28) found that aerobic exercise undertaken four times a week lessens the effect of inflammatory activities and improved anti-inflammatory markers after 6 months. The study reported improvement in glucose control, insulin resistance, and anti-inflammatory protection via increased IL-10 (4.14 ± 5.65 vs. −0.23 ± 4.73 pg/ml), and suppression of IL-18, and IL-18/IL-10 ratio (*p* > 0.05) in 60 overweight patients with T2DM who do not have vascular complications (28). Although TNF-α levels (*p* = 0.77) remained constant, hs-CRP was significantly decreased (*p* = 0.04) and was independent of significant alteration in IL-18 as the participants were reported to be on antidiabetic drugs (28). Also, systolic blood pressure was considerably improved (*p* < 0.05), while diastolic blood pressure decreased and IL-10 was elevated after the exercise program.

Exercise has been reported in previous studies to ameliorate cellular apoptosis, atherosclerotic plaque, tissue factor expression, and nuclear factor-κB activation (36–38). While these findings are revealing and an enhancement of IL-10 levels were reported in T2DM participants by Kadoglou et al. (28) after exercise training, the participants had no history of cardiovascular complications which limits the study conclusions for aerobic exercise as an intervention for protective risk outcome in diabetic angiopathy. Taken together, the study by Choi et al. (24) and Kadoglou et al. (28) reported a reduction in hs-CRP after an aerobic exercise; Choi et al. (24) found a statistically significant difference (*p* < 0.013) in the levels of hs-CRP measured at the start and end of the study, while Kadoglou et al. (28) reported a 39.6% reduction in hs-CRP (*p* < 0.04) at the end of the aerobic exercise. Choi et al. (24) also reported a statistically significant difference (*p* < 0.05) in the levels of IL-6 for the exercise group compared with the control. While participants in the study by Choi et al. (24) had both vascular and cardiometabolic risk, those in Kadoglou et al. (28) were reported to have only cardiovascular risk. The anti-inflammatory effects of exercise seemed to result in greater significant effect size as it correlated to decreased HbA1c and blood pressure, and was negatively correlated with increased soluble receptor for AGEs which suggest that aerobic exercise improved systemic inflammatory state in T2DM.

### Effect of Dietary Supplement on Measures of Inflammation in People With T2DM

Dietary supplements such as ginger powder, dietary raspberries, and hesperidin were the intervention used by Zarezadeh et al. (30), Schell et al. (26), and Homayouni et al. (25), respectively, to investigate intervention effectiveness on inflammatory activities linked with vascular complications. Schell et al. (26) and Homayouni et al. (25) found a significant reduction (*p* < 0.05) in levels of IL-6, TNF-α, and hs-TNF, while Zarezadeh et al. (30) reported that ginger supplement decreased serum ICAM-1 marginally, serum ICAM-1 has previously been associated with the pathogenesis of cardiovascular disease in T2DM. Schell et al. (26) did not find a significant difference in CRP after red raspberries were consumed, but reported that daily raspberry consumption for a period of 4 weeks showed a consistently significant lowering effect (*p* < 0.05) on IL-6 and high-sensitivity TNF-α (hs-TNF-α) at 4 h postprandial after meal.

Furthermore, hesperidin was suggested to have anti-inflammatory effects in T2DM as there was a significant difference (*p* < 0.05) in TNF-α, hs-CRP, and IL-6 inflammatory markers between the control and experimental groups of the RCT that investigated the consumption of hesperidin supplement and its effect on blood pressure and inflammatory activities on 64 T2DM patients (25). A statistically significant (*p* = 0.001) decrease in levels of TNF-α and IL-6 (*p* = 0.034) markers of inflammation, respectively, was reported, but no difference in the mean hs-CRP, rather an increased serum total antioxidant capacity (TAC) at the end in comparison with baseline values was reported. In addition, the percent change of TNF-α, IL-6, and hs-CRP levels were significantly reduced in the treated group compared with the control (*p* < 0.05), indicating that hesperidin has anti-inflammatory effects in T2DM (25). Although the study reported repeated assessment of dietary components and showed that hesperidin may improve TAC and reduce inflammation in patients with T2DM, unreported or poor dietary compliance of hesperidin supplement by the participants, the inability to measure antioxidant enzymes and serum nitric oxide (NO) level, and lack of intention-to-treat analysis could have limited the study findings (25).

The long- and short-term hesperidin administration have been reported to have anti-hypertensive effects and to stimulate NO production via phosphorylation of enzymes that mediate its output in vascular endothelial cells (39, 40). Hesperidin probably controls vascular tone by increasing vasorelaxing factors like NO or other tone modulating products such as NADPH oxidase (41, 42). It is believed that the onset of ROS disrupts NO production and leads to an increase in prostaglandin-like compounds that are suggested to have proinflammatory actions (25). The anti-inflammatory and antioxidant benefits of hesperidin are strongly correlated with hypertension and oxidative stress; (43) and it has been reported that hesperidin represses ROS-generating enzymes (43–45). It is important that ROS are repressed because inflammation in diabetic condition is induced by elevated ROS (25) which promotes the pathogenesis of cardiovascular disease in people with T2DM, and this can be measured through changes in ICAM-1 (inter-cellular adhesion molecule-1) and ADMA (asymmetric dimethylarginine) biomarkers (30).

A significant difference in ADMA serum levels (*p* = 0.002) and a marginal change in serum ICAM-1 (*p* = 0.097) were reported in the ginger supplementation group by Zarezadeh et al. (30) in the study to determine the effect of ginger supplementation on ICAM-1 serum levels and ADMA in 45 patients with T2DM after 10 weeks. In the same study, another intervention group received wheat flour, but the ADMA and serum ICAM-1 differences between the groups were not statistically significant, giving slight credence to ginger as a possible intervention. The study by Zarezadeh et al. (30) was conducted in a University hospital in Iran and did not include information on the participant's gender (30). Similarly, the study by Homayouni et al. (25) which was also conducted in a University hospital in Iran had no information on the participant's gender, but that by Schell et al. (26) carried out in a clinical assessment unit in the USA had gender information and the selected participants consisted of more males (25) than females (5).

#### Effect of Medication on Measures of Inflammation in People With T2DM

A handful of studies have investigated the anti-inflammatory effect of medication on measures of inflammation. Alkhalaf et al. (23) studied the outcome of benfotiamine intervention in atherosclerotic plaque inflammation. The investigation of benfotiamine and its effect on markers of low-grade inflammation in 39 T2DM patients with nephropathy who had high-normal urinary albumin excretion(UAE) and UAE in the microalbuminuric range (15–300 mg/24 h) found no significant change in hs-CRP, MPO, and sAA compared with the control group after 12 weeks (23). Markers of endothelial dysfunction and AGE yielded insignificant results, suggesting that inflammation-induced vascular complications in T2DM are unaffected by benfotiamine (23), although the patients were found to have had other medications such as angiotensin receptor blockers (ARBs) and ACE inhibitors which might have introduced confounding. While Alkhalaf et al. (23) found no significant difference in hs-CRP, sAA, and MPO between the benfotiamine or placebo-treated groups, Mizoguchi et al. (31) reported that hs-CRP levels decreased after pioglitazone (*p* < 0.01) treatment but increased marginally by glimepiride (*p* < 0.05), and the increasing hs-CRP levels were associated with atherosclerotic plaque inflammation. However, the small number of participants on pioglitazone (31 patients), some who were on other medications such as statins, aspirin, and renin–angiotensin system inhibitors, could potentially have introduced confounding variables that may interact with the efficacy of the intervention via drug–drug interaction (46). The short duration of the study and the fact that only atherothrombotic stroke was of interest is a limitation of the generalizability of the study with regards to diabetic angiopathies (31).

In a similar experiment to investigate the outcome of pioglitazone treatment, Mizoguchi et al. (31) found increased high-density lipoprotein (HDL) upon pioglitazone administration, though when compared with glimepiride, a stepwise multiple regression analysis considered this independent in association with inflammation-induced atherosclerosis. The attenuation of atherosclerotic plaque inflammation may be associated with increased HDL levels as previous findings reported increased HDL cholesterol levels after statin administration, but decreased plaque inflammation in dyslipidemic patients (47). HDL cholesterol promotes antioxidant, anti-inflammatory events attenuating atherosclerosis (31), and high level of HDL can reduce the odds of T2DM patients developing microvascular retinopathy and renal disease (48). In contrast, the AIM-HIGH clinical trial result found no cardiovascular benefit in 34% of its participants with T2DM despite a noticeable increase in HDL cholesterol level (49). The observation that atherosclerotic plaque inflammation–attenuating properties of pioglitazone could be largely ascribed to its HDL-increasing property rather than its hs-CRP-lowering property suggests a difference in systemic and plaque inflammation, and that hs-CRP, a marker for chronic systemic inflammation, may not reflect plaque inflammation (31). Nevertheless, the study suggests that pioglitazone could be a potent intervention in attenuating inflammation-induced atherosclerosis.

The findings on salsalate treatment and the outcome from combined treatment with simvastatin and fenofibrate were insightful and beneficial. Barzilay et al. (27) in a 48-week TINSAL-T2D study found that salsalate moderated hyperglycemia and serum levels of AGEs which are involved in diabetes-related vascular complications after 118 out of 207 total participants received 3.5 g/day of salsalate (27). A noticeable significant change (*p* > 0.05) in WBC and furosine but not in other glycation products for CRP and TNF in salsalate users was reported (27). Although Barzilay et al. (27) stated that the reasons for not detecting a change in serum-protein-bound AGE levels were not exactly known, the fact that AGE could have multiple pathways, that metformin was taken by most participants, and that the salsalate-treated participants with hypoglycemic events were receiving reduced antihyperglycemic medications were suggested as a possible explanation. Barzilay et al. (27) found that salsalate had no effect on late AGEs but reduced early AGEs and had a significant lowering effect on HbA1c; however, there was an increase in serum pentosidine (an AGE) indicating the possibility of induced oxidative stress, while changes in markers of inflammation or renal factors were independent of changes in pentosidine and glycation factors.

Interestingly, Krysiak et al. (29) in a separate study found a significant decrease (*p* = 0.001) in all of TNF, IL-1, IL-2, IL-6, MCP-1, and CRP inflammatory activities after a combined treatment with simvastatin and fenofibrate, but a weak correlation (*r* = 0.20) with single fenofibrate administration. The study, which involved 196 T2DM participants with mixed dyslipidemia, reported that the medications exerted a similar but more efficacious effect on systemic inflammation after combined administration (29). However, single administration of simvastatin reduced monocyte release of pro-inflammatory cytokines such as TNF-α, IL-1β, IL-6, and monocyte chemoattractant protein-1 (MCP-1); and lymphocyte release of IL-2, interferon-γ, and TNF-α; including a decrease in plasma CRP levels, while single administration of fenofibrate had anti-inflammatory effects that correlated with improved insulin sensitivity and downregulation of lymphocytes rather than monocytes (29). Lymphocytes and monocytic cells are present in atherosclerotic plaques and are very well associated with the progression of atherosclerosis (50–52).

Assessment of the RCTs using medication as an intervention showed that none of the four studies were multi-country and majority of the selected participants were males; for example, the RCT by Alkhalaf et al. (23) was conducted in an outpatient clinic in the Netherlands and all participants were males which limits the conclusion for females. The representation of gender was skewed toward the male in the other studies; for example, selected participants in the study by Krysiak et al. (29) had 108 males and 82 females. Also, Barzilay et al. (27) had 130 male and 97 female participants, while there were 39 males and 13 females in the study by Mizoguchi et al. (31). All participants had T2DM; however, participants in the study by Alkhalaf et al. (23) also had UAE, while those in the RCT conducted by Krysiak et al. (29) had untreated T2DM. These additional disorders may represent confounding factors which may not have been accounted for by the RCTs.

## Discussion

Diabetic angiopathies can be induced by low-grade inflammation, AGEs, and endothelial dysfunction which are considered intermediate pathways (23). TNF-α, IL-1β, IL-6, MCP-1, IL-2, and interferon-γ are some of the monocyte- and lymphocyte-derived pro-inflammatory cytokines (29); these cytokines as well as CRP are markers of chronic low-grade systemic inflammation and prognostic indicators of vascular complications and increased cardiovascular risk (29). The risk for vascular complications and the extent of insulin resistance have been linked with changes in the levels of these inflammatory markers (53–55). Inflammation just like hyperglycemia, is an integral process leading to atherosclerosis and increased risk of atherosclerotic cardiovascular disease (31). However, there is little evidence to suggest that a particular oral anti-diabetic medication is the preferred intervention to ameliorate inflammation-induced atherosclerosis or angiopathies (31). Drugs such as pioglitazone, simvastatin, and fenofibrate, and non-acetylated salicylate such as salsalate but not benfotiamine suggested benefits in moderating vascular complications (23, 27, 29, 31).

Benfotiamine had been previously suggested as a therapy for diabetic nephropathy and other related complications through its activation of transketolase for the inhibition of AGE precursors, low-grade inflammation, and endothelial dysfunction in an experiment using animals with diabetes (56, 57). Transketolase, an enzyme involved in the breakdown of glucose, plays a role in inhibiting vascular complications (58, 59), and although Alkhalaf et al. (23) reported a significant increase in transketolase activity (a co-factor of thiamine), no significant findings from benfotiamine administration across the two study groups were observed, suggesting that further long-term studies are needed to understand the pathway and mechanisms of its action in humans (56). The study by Alkhalaf et al. (23) was one of the few papers that reported findings on measures of inflammatory markers after benfotiamine administration.

Nonetheless, the TINSAL-T2D study found that salsalate treatment did not overly change most AGE levels (27). However, pioglitazone was found to ameliorate inflammation-induced atherosclerosis in T2DM. Mizoguchi et al. (31) evaluating the effect of 0.5–4.0 mg glimepiride and 15–30 mg pioglitazone intervention on atherosclerotic plaque inflammation after 4 months, found that both interventions decreased HbA1c, but only pioglitazone ameliorated inflammation-induced atherosclerosis. This result agreed with the PERISCOPE and CHICAGO studies that showed that when compared with glimepiride, pioglitazone prevented the continuous development of carotid or coronary atherosclerosis after 18 months of treatment (60, 61), providing evidence for pioglitazone as a protective risk factor for diabetes-related angiopathies. Furthermore, the use of simvastatin, a drug with anti-inflammatory properties (29, 62, 63), and fenofibrate was found to be a clinically relevant medication in the treatment of low-grade inflammation for the prevention of atherosclerosis (29).

Nevertheless, there are contrasting findings on fenofibrate intervention in other studies. For example, the FIELD and BIP trial studies on lowering events in diabetes found that fibrate significantly decreased cardiovascular risk by only 19 and 17%, respectively; although there was a decrease in mortality and nonfatal heart attack, the result was not significant (64). In addition, the Action to Control Cardiovascular Risk in Diabetes Lipid trial (ACCORD) after a 10-year study reported decreased cerebrovascular risks in middle-aged and older T2DM patients using simvastatin intervention alone or in combination with fenofibrate (65). The difference between the findings by Krysiak et al. (29) and the ACCORD trial was on the superior potency of the combination of simvastatin and fenofibrate compared with monotherapy. The ACCORD trial highlighted the potency of simvastatin as well as in combination with fenofibrate, while Krysiak et al. (29) reported that both combined and monotherapy administration of simvastatin and fenofibrate equally affected monocyte cytokine release, but only the combined medication decreased lymphocyte cytokine release and improved lipid profile. These differences could be associated with the characteristics of the participants as Krysiak et al. (29) included lifestyle modification and metformin in the study of a small number of newly diagnosed patients with T2DM who also had dyslipidemia, and administered the combined therapy at reduced dosage for a shorter period, unlike the ACCORD trial that was a 10-year study.

The role of systemic inflammation in T2DM-related vascular complications have been linked with low levels of soluble receptor for AGEs (24) and chronic inflammation which is correlated with increasing risk for cardiovascular events (28). Soluble RAGE was suggested to ameliorate the adverse and inflammatory action of RAGE, and patients with T2DM have been reported to have low levels of circulating soluble RAGE (66, 67). The circulating soluble RAGE has an indirect relationship with insulin resistance, HbA1c, and CRP, making it a marker and predictor for inflammation and cardiovascular events (68, 69). The report on lower systemic inflammation due to the induction of soluble RAGE in patients with T2DM by Choi et al. (24) is consistent with other studies by Arikawa et al. (70), Nadar and Lundberg (71), and Riesco et al. (72) all of whom reported significant decrease in CRP and IL-6 levels after 4–6 months of regular exercise. The precise fundamental process responsible for generating soluble RAGE via regular exercise remains unclear (24).

Accordingly, pro-inflammatory markers such as IL-18 are elevated in diabetes and strongly considered as an independent predictor of adverse cardiovascular events (73, 74). The alterations in IL-18 were positively linked with changes in insulin resistance in one of the selected studies (28), and other studies have reported changes in IL-18 levels influenced by lifestyle modification such as exercise (28, 75). The process in which exercise lowers IL-18 concentration is probably by modifying insulin signaling or downregulating cytokine production decreasing inflammatory cell infiltration of adipose tissues (76); however, determining the association of the cause and effect still remains unclear (28). In contrast, anti-inflammatory serum levels of IL-10 secreted by T cells of Th2 subtype, macrophages, and monocytes in humans have been shown to cancel out the risk of cardiac events (33).

Inflammation independently induces the risk for hypertension (25, 77, 78). Modern diets are said to be replete with AGEs which are generated during food processing and have been implicated in the pathogenesis of diabetes-related disorders due to their ability to activate chronic low-grade inflammation and insulin resistance (79). The speed at which AGEs increase in amount in the body differs among people and is dependent on lifestyle such as cigarette smoking, consumption of AGE-rich diet, age, and metabolic health of an individual (79). The consumption of AGE-rich diet (e.g., fried meats, glycated proteins, toasted wheat flakes, etc.) leads to the proliferation of pathogenic colonic bacteria; therefore, eating healthy diets low in AGE may reduce AGE accumulation from exogenous sources. Yet, the difficulty is in resisting and overcoming the temptation to consume those meals as AGE-rich diets have enhanced aroma, flavor, and color (80). One solution has been the supplementation with prebiotic fibers which improve optimal gut microbial balance to inhibit AGE in people at risk of T2DM (79). Prebiotic fibers such as inulin and raspberries could reduce the glycemic index of a diet, slow the absorption of intestinal nutrient, and delay gastric emptying (79).

Dietary interventions geared at preventing the progression of inflammatory activities are necessary to attenuate diabetes-related angiopathies. Hesperidin, ginger, and dietary raspberry supplementation from the selected studies were suggested as dietary supplements that could ameliorate the effect of inflammation and AGEs (25, 26, 30). Hesperidin inhibits the production of inflammatory cytokines that increase arterial stiffness and peripheral vascular resistance which are indications for hypertension and vascular complications (24). Raspberries are prebiotic fibers whose products may lower the absorption of exogenous AGE in the gut and inhibit circulating proinflammatory cytokines; they can also induce changes that enhance gut barrier functioning by elevating the production of glucagon-like peptide 2 (GLP-2) which promotes an increase in crypt cells (79, 81, 82). Food supplements like ginger have been reported to have anti-inflammatory and anti-oxidant properties (30) which lowers ADMA, which when elevated decreases NO production leading to decreased blood supply to peripheral tissues (30).

An increase in ADMA promotes the activation of nuclear factor kappa B (NF-κB) transcription factor which is associated with increased ICAM-1 and proinflammatory cytokine production (30). ICAM-1 is a protein expressed through NF-κB pathway in endothelial cells and leukocytes (30). ADMA makes the NF-κB pathway active, leading to increased ICAM-1 (83). ADMA and ICAM-1 play a role in diabetes-related cardiovascular complications and insulin resistance level as they are involved in pathway association with oxidative stress, NO inhibition, increased blood pressure, and inflammation (84–87). During inflammatory conditions, leukocytes produce and attach ICAM-1 protein to endothelial cells promoting macrophage production of foam cells (88). Therefore, inhibition of NF-κB transcription factor, ADMA, and ICAM-1 could prevent cardiovascular complications in T2DM (30).

Taken all together, there was limited information on participant compliance during the interventions. Also, not all studies included information on gender and disclosed the current medication in the baseline characteristics of the participants at the beginning and end of the study, especially because the participants were known T2DM patients. Most studies had more male participants and were not multi-country, implying lack of diversity in racial origin and ethnicity which could potentially limit the representation of the conclusions. It is important to note that the selected studies looked at interventions on inflammatory activities in people with diabetes. Moreover, the data selection was done by a single reviewer but verified by another reviewer for consistency. The small sample size of participants and limited RCTs on inflammatory activities in T2DM-related comorbidities is a limitation. However, the study contributes to medical knowledge and education for carers and people with diabetes-related angiopathies by reviewing information on how intake of healthy dietary supplements, aerobic exercise, and anti-inflammatory medications can improve protection from future risk by highlighting beneficial interventions that may lower the potential for negative outcomes.

## Conclusion

Elevated inflammatory activities and its association pathways such as endothelial dysfunction and AGEs have been reported in diabetes-related vascular disorders. TNF-α and IL-6 were the most common proinflammatory cytokines from the selected studies; together with CRP, their alterations represent an important biomarker for accessing inflammatory activities in diabetic angiopathies. The activities of IL-10, an anti-inflammatory cytokine, is helpful in accessing reduced risk for induced inflammation in diabetes-related angiopathies. Regular aerobic exercise, medications that decrease proinflammatory activities, as well as diet rich in prebiotic fibers and those possessing anti-inflammatory properties were suggested as a means of attenuating the risk of increased proinflammatory events.

A summary of knowledge from the findings suggested that salsalate treatment could reduce inflammatory activity but may not change AGE levels which is linked to oxidative stress because markers of serum inflammation only indirectly reflect oxidative stress. The single administration of simvastatin was found to downregulate monocytic and lymphocytic release of proinflammatory markers, whereas fenofibrate only reduced markers of lymphocytic inflammation. The administration of both drugs were suggested to halt the progression of diabetes-related atherosclerosis because lymphocytes and monocytic cells are present in atherosclerotic plaques. Similarly, pioglitazone was found to prevent the continuous progression of inflammation-induced atherosclerosis by increasing HDL and lowering hs-CRP levels, indicating its involvement in reducing systemic and plaque inflammation.

Finally, while it is known that changes in metabolic status and cytokines could increasingly alter insulin action, and that exercise may lower cellular apoptosis and atherosclerotic plaque, not much of this has been studied with regards to people with T2DM and existing angiopathies or on the effectiveness of various food supplements. In this study, aerobic exercise was shown to reduce risk factors associated with vascular and cardiometabolic risk by increasing soluble RAGE which interacts with AGE, a known promoter of atherosclerosis in diabetes condition. Indeed, the review showed that regular exercise increases anti-inflammatory activities and reduces proinflammatory events and mediators of oxidative stress such as AGE which is high in AGE-rich diets. Prebiotic fibers such as raspberries lower the absorption of AGEs in the gut, while ginger and hesperidin lower inflammatory activities that promote cardiovascular and vascular complications by suppressing ADMA, regulating ICAM-1, and stimulating DDAH and NO production. Thus, medication and healthy dietary supplements with anti-inflammatory properties, along with regular exercise, may result in beneficial outcome for diabetes patients.

## Data Availability Statement

The original contributions presented in the study are included in the article/Supplementary Material, further inquiries can be directed to the corresponding author/s.

## Author Contributions

MN devised the conceptual ideas and wrote the article.

## Conflict of Interest

The author declares that the research was conducted in the absence of any commercial or financial relationships that could be construed as a potential conflict of interest.

## Abbreviations

AIM-HIGH: Atherothrombosis Intervention in Metabolic Syndrome with Low HDL/High Triglycerides: Impact on Global Health Outcomes Trial
HbA_1c_: hemoglobin A_1c_
hs-CRP: high-sensitivity C reactive protein
TNF-α: umor necrosis factor-alpha
IL: interleukin
sRAGE: soluble receptor for advanced glycation end-products
sAA: serum amyloid A
ICAM-1: intercellular adhesion molecule 1
MPC-1: monocyte chemoattractant protein-1
WBC: white blood cell
TAC: total antioxidant capacity
MPO: myeloperoxidase
T2DM: type 2 diabetes mellitus
T1DM: type 1 diabetes mellitus
TINSAL-T2D: Targeting Inflammation Using Salsalate for Type 2 Diabetes.
