# Supplementary material for: Inflammatory Activities in Type 2 Diabetes Patients With Co-morbid Angiopathies and Exploring Beneficial Interventions: A Systematic Review
Source: Front Public Health. 2021 Jan 25;8:600427. doi: 10.3389/fpubh.2020.600427 (PMC7868423; doi:10.3389/fpubh.2020.600427)
Supplement: Supplementary file 1 [file Table_1.docx]

**Appendices**

Appendix 1: Pubmed Queries

Appendix 2: Ovid Medline Queries

Appendix 3: Embase Query

Appendix 1: Pubmed Queries

|  | **Pubmed queries** |  |
| --- | --- | --- |
| **Search** | **Query** | **Items found** |
| #4 | Search ("Type 2 diabetes" OR "Type II diabetes" OR "Diabetes-Mellitus-Non-Insulin-Dependent" OR "diabetes type 2" OR "Adult Onset Diabetes Mellitus" OR "Slow Onset Diabetes Mellitus" OR "Adult-Onset Diabetes Mellitus" OR "Slow-Onset Diabetes Mellitus" OR "Stable Diabetes Mellitus" OR "Ketosis Resistant Diabetes Mellitus") Sort by: [pubsolr12] | 179274 |
| #3 | Search ("Diabetic Microangiopathy" OR "Microangiopathies Diabetic" OR "Diabetic Microangiopathies" OR "Vascular Diseases Diabetic" OR "Angiopathies Diabetic" OR "Diabetic Vascular Complication" OR "Diabetic Vascular Disease" OR "Vascular Complications" OR "Diabetic Vascular Disease" OR "Diabetic Vascular Diseases" OR "Diabetic Vascular Complications") Sort by: [pubsolr12] | 14951 |
| #2 | Search (Inflammation* OR "Acute-Phase Reaction" OR "Foreign-Body Reaction" OR "Neurogenic Inflammation" OR Seroma* OR "Serositis" OR Suppuration* OR "Systemic Inflammatory Response Syndrome") Sort by: [pubsolr12] | 567008 |
| #1 | Search ((("Type 2 diabetes" OR "Type II diabetes" OR "Diabetes-Mellitus-Non-Insulin-Dependent" OR "diabetes type 2" OR "Adult Onset Diabetes Mellitus" OR "Slow Onset Diabetes Mellitus" OR "Adult-Onset Diabetes Mellitus" OR "Slow-Onset Diabetes Mellitus" OR "Stable Diabetes Mellitus" OR "Ketosis Resistant Diabetes Mellitus")) AND ("Diabetic Microangiopathy" OR "Microangiopathies Diabetic" OR "Diabetic Microangiopathies" OR "Vascular Diseases Diabetic" OR "Angiopathies Diabetic" OR "Diabetic Vascular Complication" OR "Diabetic Vascular Disease" OR "Vascular Complications" OR "Diabetic Vascular Disease" OR "Diabetic Vascular Diseases" OR "Diabetic Vascular Complications")) AND (Inflammation* OR "Acute-Phase Reaction" OR "Foreign-Body Reaction" OR "Neurogenic Inflammation" OR Seroma* OR "Serositis" OR Suppuration* OR "Systemic Inflammatory Response Syndrome") Sort by: [pubsolr12] | 219 |

Appendix 2: Ovid Medline Queries

|  | **Ovid Medline queries** |  |
| --- | --- | --- |
| **#** | **Searches** | **Results** |
| 1 | "Type 2 diabetes".mp. [mp=title, abstract, original title, name of substance word, subject heading word, floating sub-heading word, keyword heading word, organism supplementary concept word, protocol supplementary concept word, rare disease supplementary concept word, unique identifier, synonyms] | 121841 |
| 2 | Type II diabetes$.mp. [mp=title, abstract, original title, name of substance word, subject heading word, floating sub-heading word, keyword heading word, organism supplementary concept word, protocol supplementary concept word, rare disease supplementary concept word, unique identifier, synonyms] | 8242 |
| 3 | Diabetes-Mellitus-Non-Insulin-Dependent$.mp. [mp=title, abstract, original title, name of substance word, subject heading word, floating sub-heading word, keyword heading word, organism supplementary concept word, protocol supplementary concept word, rare disease supplementary concept word, unique identifier, synonyms] | 33 |
| 4 | diabetes type 2$.mp. [mp=title, abstract, original title, name of substance word, subject heading word, floating sub-heading word, keyword heading word, organism supplementary concept word, protocol supplementary concept word, rare disease supplementary concept word, unique identifier, synonyms] | 1358 |
| 5 | Adult Onset Diabetes Mellitus$.mp. [mp=title, abstract, original title, name of substance word, subject heading word, floating sub-heading word, keyword heading word, organism supplementary concept word, protocol supplementary concept word, rare disease supplementary concept word, unique identifier, synonyms] | 145 |
| 6 | Slow Onset Diabetes Mellitus$.mp. [mp=title, abstract, original title, name of substance word, subject heading word, floating sub-heading word, keyword heading word, organism supplementary concept word, protocol supplementary concept word, rare disease supplementary concept word, unique identifier, synonyms] | 0 |
| 7 | Adult-Onset Diabetes Mellitus$.mp. [mp=title, abstract, original title, name of substance word, subject heading word, floating sub-heading word, keyword heading word, organism supplementary concept word, protocol supplementary concept word, rare disease supplementary concept word, unique identifier, synonyms] | 145 |
| 8 | Slow-Onset Diabetes Mellitus$.mp. [mp=title, abstract, original title, name of substance word, subject heading word, floating sub-heading word, keyword heading word, organism supplementary concept word, protocol supplementary concept word, rare disease supplementary concept word, unique identifier, synonyms] | 0 |
| 9 | Stable Diabetes Mellitus$.mp. [mp=title, abstract, original title, name of substance word, subject heading word, floating sub-heading word, keyword heading word, organism supplementary concept word, protocol supplementary concept word, rare disease supplementary concept word, unique identifier, synonyms] | 16 |
| 10 | Ketosis Resistant Diabetes Mellitus$.mp. [mp=title, abstract, original title, name of substance word, subject heading word, floating sub-heading word, keyword heading word, organism supplementary concept word, protocol supplementary concept word, rare disease supplementary concept word, unique identifier, synonyms] | 1 |
| 11 | 1 or 2 or 3 or 4 or 5 or 6 or 7 or 8 or 9 or 10 | 130433 |
| 12 | Diabetic Microangiopathy$.mp. [mp=title, abstract, original title, name of substance word, subject heading word, floating sub-heading word, keyword heading word, organism supplementary concept word, protocol supplementary concept word, rare disease supplementary concept word, unique identifier, synonyms] | 1192 |
| 13 | Microangiopathies Diabetic$.mp. [mp=title, abstract, original title, name of substance word, subject heading word, floating sub-heading word, keyword heading word, organism supplementary concept word, protocol supplementary concept word, rare disease supplementary concept word, unique identifier, synonyms] | 1 |
| 14 | Diabetic Microangiopathies$.mp. [mp=title, abstract, original title, name of substance word, subject heading word, floating sub-heading word, keyword heading word, organism supplementary concept word, protocol supplementary concept word, rare disease supplementary concept word, unique identifier, synonyms] | 109 |
| 15 | Vascular Diseases Diabetic$.mp. [mp=title, abstract, original title, name of substance word, subject heading word, floating sub-heading word, keyword heading word, organism supplementary concept word, protocol supplementary concept word, rare disease supplementary concept word, unique identifier, synonyms] | 4 |
| 16 | Angiopathies Diabetic$.mp. [mp=title, abstract, original title, name of substance word, subject heading word, floating sub-heading word, keyword heading word, organism supplementary concept word, protocol supplementary concept word, rare disease supplementary concept word, unique identifier, synonyms] | 1 |
| 17 | Diabetic Vascular Complication$.mp. [mp=title, abstract, original title, name of substance word, subject heading word, floating sub-heading word, keyword heading word, organism supplementary concept word, protocol supplementary concept word, rare disease supplementary concept word, unique identifier, synonyms] | 862 |
| 18 | Diabetic Vascular Disease$.mp. [mp=title, abstract, original title, name of substance word, subject heading word, floating sub-heading word, keyword heading word, organism supplementary concept word, protocol supplementary concept word, rare disease supplementary concept word, unique identifier, synonyms] | 379 |
| 19 | Vascular Complications$.mp. [mp=title, abstract, original title, name of substance word, subject heading word, floating sub-heading word, keyword heading word, organism supplementary concept word, protocol supplementary concept word, rare disease supplementary concept word, unique identifier, synonyms] | 10528 |
| 20 | Diabetic Vascular Disease$.mp. [mp=title, abstract, original title, name of substance word, subject heading word, floating sub-heading word, keyword heading word, organism supplementary concept word, protocol supplementary concept word, rare disease supplementary concept word, unique identifier, synonyms] | 379 |
| 21 | Diabetic Vascular Diseases$.mp. [mp=title, abstract, original title, name of substance word, subject heading word, floating sub-heading word, keyword heading word, organism supplementary concept word, protocol supplementary concept word, rare disease supplementary concept word, unique identifier, synonyms] | 43 |
| 22 | Diabetic Vascular Complications$.mp. [mp=title, abstract, original title, name of substance word, subject heading word, floating sub-heading word, keyword heading word, organism supplementary concept word, protocol supplementary concept word, rare disease supplementary concept word, unique identifier, synonyms] | 828 |
| 23 | 1 or 2 or 3 or 4 or 5 or 6 or 7 or 8 or 9 or 10 or 11 or 12 or 13 or 14 or 15 or 16 or 17 or 18 or 19 or 20 or 21 or 22 | 141255 |
| 24 | Inflammation*.mp. [mp=title, abstract, original title, name of substance word, subject heading word, floating sub-heading word, keyword heading word, organism supplementary concept word, protocol supplementary concept word, rare disease supplementary concept word, unique identifier, synonyms] | 507114 |
| 25 | Acute-Phase Reaction$.mp. [mp=title, abstract, original title, name of substance word, subject heading word, floating sub-heading word, keyword heading word, organism supplementary concept word, protocol supplementary concept word, rare disease supplementary concept word, unique identifier, synonyms] | 4137 |
| 26 | Foreign-Body Reaction$.mp. [mp=title, abstract, original title, name of substance word, subject heading word, floating sub-heading word, keyword heading word, organism supplementary concept word, protocol supplementary concept word, rare disease supplementary concept word, unique identifier, synonyms] | 7608 |
| 27 | Neurogenic Inflammation$.mp. [mp=title, abstract, original title, name of substance word, subject heading word, floating sub-heading word, keyword heading word, organism supplementary concept word, protocol supplementary concept word, rare disease supplementary concept word, unique identifier, synonyms] | 2380 |
| 28 | Seroma*.mp. [mp=title, abstract, original title, name of substance word, subject heading word, floating sub-heading word, keyword heading word, organism supplementary concept word, protocol supplementary concept word, rare disease supplementary concept word, unique identifier, synonyms] | 5491 |
| 29 | Serositis$.mp. [mp=title, abstract, original title, name of substance word, subject heading word, floating sub-heading word, keyword heading word, organism supplementary concept word, protocol supplementary concept word, rare disease supplementary concept word, unique identifier, synonyms] | 1389 |
| 30 | Suppuration*.mp. [mp=title, abstract, original title, name of substance word, subject heading word, floating sub-heading word, keyword heading word, organism supplementary concept word, protocol supplementary concept word, rare disease supplementary concept word, unique identifier, synonyms] | 10282 |
| 31 | Systemic Inflammatory Response Syndrome$.mp. [mp=title, abstract, original title, name of substance word, subject heading word, floating sub-heading word, keyword heading word, organism supplementary concept word, protocol supplementary concept word, rare disease supplementary concept word, unique identifier, synonyms] | 8155 |
| 32 | 25 or 26 or 27 or 28 or 29 or 30 or 31 or 32 | 538190 |
| 33 | 11 and 24 and 33 | 150 |
| 34 | limit 35 to (english language and humans) | 104 |

Appendix 3: Embase Query

| **No.** | **Embase Query** | **Results** |
| --- | --- | --- |
| #4 | #2 AND #3 AND #4 | 131 |
| #3 | ('type 2 diabetes'/exp OR 'type 2 diabetes' OR 'type ii diabetes'/exp OR 'type ii diabetes' OR 'diabetes-mellitus-non-insulin-dependent'/exp OR 'diabetes-mellitus-non-insulin-dependent' OR   'diabetes type 2'/exp OR 'diabetes type 2' OR 'adult onset diabetes mellitus'/exp OR 'adult onset diabetes mellitus' OR 'slow onset diabetes mellitus' OR 'adult-onset diabetes mellitus'/exp OR 'adult-onset diabetes mellitus' OR 'slow-onset diabetes mellitus' OR 'stable diabetes mellitus' OR 'ketosis resistant diabetes mellitus'/exp OR 'ketosis resistant diabetes mellitus') AND [humans]/lim AND [clinical study]/lim AND [embase]/lim | 123834 |
| #2 | ('diabetic microangiopathy'/exp OR 'diabetic microangiopathy' OR 'microangiopathies diabetic' OR 'diabetic microangiopathies' OR 'vascular diseases diabetic' OR 'angiopathies diabetic' OR 'diabetic vascular complication' OR 'vascular complications' OR 'diabetic vascular disease' OR 'diabetic vascular diseases' OR 'diabetic vascular complications') AND [humans]/lim AND [clinical study]/lim AND [embase]/lim | 9687 |
| #1 | (inflammation* OR 'acute-phase reaction'/exp OR 'acute-phase reaction' OR 'foreign-body reaction'/exp OR 'foreign-body reaction' OR 'neurogenic inflammation'/exp OR 'neurogenic inflammation' OR seroma* OR 'serositis'/exp OR 'serositis' OR suppuration* OR 'systemic inflammatory response syndrome'/exp OR 'systemic inflammatory response syndrome') AND [article]/lim AND [humans]/lim AND [embase]/lim | 1211662 |
